# 3D bioprinted hydrogel/polymer scaffold with factor delivery and mechanical support for growth plate injury repair

**DOI:** 10.3389/fbioe.2023.1210786

**Published:** 2023-05-31

**Authors:** Minjie Fan, Lei Qiang, Yiwei Wang, Yihao Liu, Hanjie Zhuang, Ruoyi Guo, Yulong Ben, Qiang Li, Pengfei Zheng

**Affiliations:** ^1^ Department of Orthopaedic Surgery, Children’s Hospital of Nanjing Medical University, Nanjing, Jiangsu, China; ^2^ Key Laboratory of Advanced Technologies of Materials (MOE), School of Materials Science and Engineering, Southwest Jiaotong University, Chengdu, Sichuan, China; ^3^ Shanghai Key Laboratory of Orthopaedic Implant, Department of Orthopaedic Surgery, Shanghai Ninth People’s Hospital, Shanghai Jiao Tong University School of Medicine, Shanghai, China

**Keywords:** tissue engineering, 3D bioprinting scaffold, growth plate injury, PTH(1–34), mechanical support

## Abstract

**Introduction:** Growth plate injury is a significant challenge in clinical practice, as it could severely affect the limb development of children, leading to limb deformity. Tissue engineering and 3D bioprinting technology have great potential in the repair and regeneration of injured growth plate, but there are still challenges associated with achieving successful repair outcomes.

**Methods:** In this study, GelMA hydrogel containing PLGA microspheres loaded with chondrogenic factor PTH(1–34) was combined with BMSCs and Polycaprolactone (PCL) to develop the PTH(1–34)@PLGA/BMSCs/GelMA-PCL scaffold using bio-3D printing technology.

**Results:** The scaffold exhibited a three-dimensional interconnected porous network structure, good mechanical properties, biocompatibility, and was suitable for cellchondrogenic differentiation. And a rabbit model of growth plate injury was appliedto validate the effect of scaffold on the repair of injured growth plate. The resultsshowed that the scaffold was more effective than injectable hydrogel in promotingcartilage regeneration and reducing bone bridge formation. Moreover, the addition ofPCL to the scaffold provided good mechanical support, significantly reducing limbdeformities after growth plate injury compared with directly injected hydrogel.

**Discussion:** Accordingly, our study demonstrates the feasibility of using 3D printed scaffolds for treating growth plate injuries and could offer a new strategy for the development of growth plate tissue engineering therapy.

## 1 Introduction

The growth plate, a specialized cartilage located between the epiphysis and diaphysis of long bones in children, plays a pivotal role in controlling longitudinal bone growth, which is essential for skeletal development (Yu et al., 2022). Despite being a temporary tissue, its importance in bone elongation cannot be overstated (Tiffany and Harley, 2022). Regrettably, the growth plate shares some anatomical characteristics with the articular cartilage, such as being avascular and lacking nerve supply, thereby limiting its intrinsic self-repair capacity. Trauma or injury to this region may lead to the formation of undesirable bone bridge, causing angular deformities, limb shortening, and other serious complications (Muruganandan et al., 2022). Currently, the clinical treatment options for growth plate injuries are limited to excision and implantation of autologous fat in cases where the bone bridge accounts for less than 33%, and invasive surgery in cases where the bone bridge is larger (Yu et al., 2022). However, the therapeutic efficacy of these methods is limited and cannot fully restore the function of the growth plate, which is essential for promoting endochondral ossification and maintaining normal bone growth (Clark et al., 2015). The emergence of tissue engineering and regenerative medicine offers novel approaches to repairing injured growth plate cartilage, presenting a promising opportunity for the development of effective treatments for growth plate injuries.

Tissue engineering has emerged as a promising strategy for regenerating growth plate tissue and promoting functional recovery after injury (Shaw et al., 2018; Wang et al., 2021). With the advancement of 3D bioprinting technology, it has become possible to fabricate tissue-engineered growth plates that possess regenerative activity by incorporating seed cells and growth factors into scaffolds (Xian and Foster, 2006; Brochhausen et al., 2009; Wang et al., 2021). Structurally, the growth plate can be divided into four zones: the resting zone, proliferative zone, hypertrophic zone, and ossification zone. In the resting zone, chondrocytes slowly proliferate and predominantly produce chondrocytes. In the proliferative and hypertrophic zones, chondrocytes organize themselves into a columnar structure parallel to the long axis of the bone, undergo rapid proliferation and differentiation. Finally, in the ossification zone, chondrocytes undergo apoptosis or transdifferentiation, while blood vessels and osteoblasts infiltrate and replace the original cartilage with newly formed bone tissue (Lui, 2020). The growth plate is subject to complex mechanical environments, and excessive load can impede bone deposition. Therefore, tissue-engineered scaffolds with adequate mechanical support can help prevent the collapse of the defect area while also providing a suitable micro-environment for cell proliferation and differentiation. 3D bioprinted scaffolds can mimic the layered and columnar arrangement structure of natural growth plate cartilage, thereby enhancing the regenerative repair potential of tissue engineering in growth plate injury. Polycaprolactone (PCL) has become a popular material for cartilage tissue engineering due to its low-melting temperature, mechanical strength, biodegradability and biocompatibility (Backes et al., 2022). Previous studies have utilized 3D-printed PCL scaffolds to provide mechanical support for the repair of articular cartilage injuries (Jang et al., 2020; Li et al., 2021). Nevertheless, the potential of PCL scaffolds in providing mechanical support and facilitating repair in growth plate injuries remains unknown.

The parathyroid hormone-related protein (PTHrP) - Indian hedgehog (Ihh) signaling pathway plays a crucial role in regulating chondrocyte proliferation and hypertrophy in the growth plate, serving as an essential negative feedback axis (Long et al., 2001; Long and Ornitz, 2013; Kozhemyakina et al., 2015). Specifically, PTHrP, which is secreted by chondrocytes located in the resting zone of the growth plate, diffuses downward to act on the PTH/PTHrP receptors expressed by proliferating chondrocytes, ultimately delaying their hypertrophy (Chen et al., 2006; Chau et al., 2011). Ihh secreted by hypertrophic chondrocytes promotes chondrocyte differentiation into hypertrophic chondrocytes in a PTHrP-independent manner and also has the potential of inducing PTHrP expression. Deng et al. demonstrated that grafting PTH onto SF (SF-PTH) could effectively inhibit chondrocyte hypertrophy, facilitate transparent chondrocyte matrix formation, and serve as a useful component in constructing a biphasic scaffold for bone and cartilage defects using 3D bioprinting technology (Deng et al., 2021). Thus, by regulating the PTHrP/Ihh signaling axis, it may be possible to modulate chondrocyte proliferation and differentiation, enable regeneration of growth plate cartilage, and facilitate repair of injured growth plate. Poly (lactic acid-co-glycolic acid) (PLGA) is a biodegradable polymer that has been widely used in the field of drug delivery due to its good biocompatibility and tunable biodegradation kinetics. PLGA-based microspheres have been extensively studied for their potential to deliver a variety of therapeutic agents, including anticancer drugs, protein and peptide drugs, bacterial and viral DNA, etc. The use of PLGA as a carrier for drug delivery has several advantages, such as sustained release of the drug, protection of the drug from degradation, improved solubility, and increased bioavailability. Moreover, Gelatin methacryloyl (GelMA) hydrogels have shown promising potential in tissue engineering due to their biomimetic properties resembling the extracellular matrix (ECM) and excellent biocompatibility (Yue et al., 2015; Guan et al., 2022). These properties make them an attractive option for various applications in tissue engineering, such as bone and cartilage regeneration.

**FIGURE 1:**
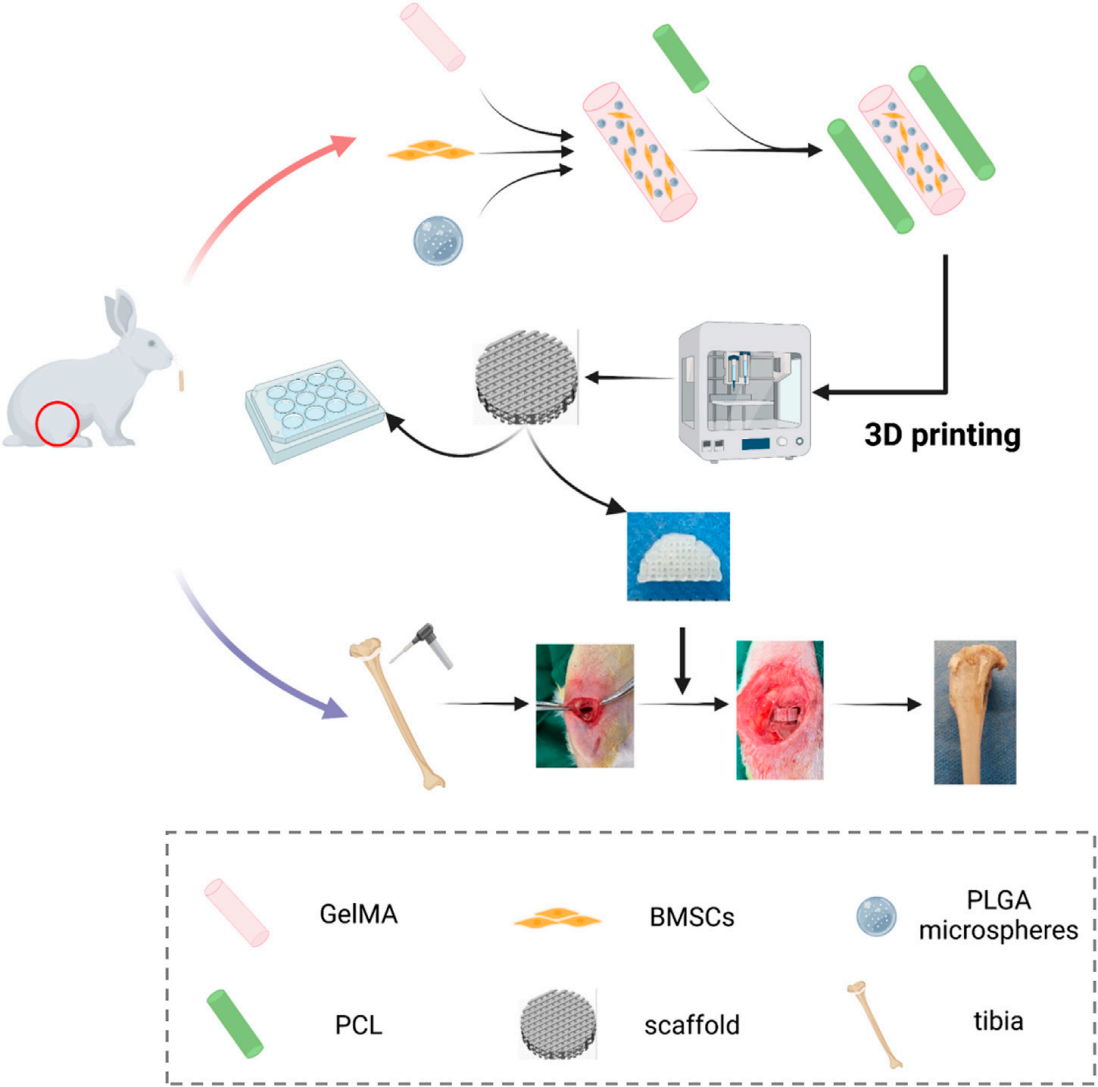
Schematic diagram of the study of PTH(1–34)@PLGA/BMSCs/GelMA-PCL scaffold.

Herein, we present the development of a 3D bioprinted growth plate composite scaffold for repairing growth plate injuries. The scaffold provides a suitable regenerative micro-environment for growth plate cartilage and rescues the formation of deformities after growth plate injury, as demonstrated in Figure 1. To achieve this, we employed a strategy of loading PTH(1–34) into PLGA microspheres and mixing it with bone marrow mesenchymal stem cells (BMSCs) and GelMA to create a “concrete” bioink. Additionally, we used PCL as “rebar” to enhance the mechanical strength of the scaffold and printed it using a dual-nozzle 3D bioprinter. We conducted *in vitro* chondrogenic performance evaluations and compared it with untreated and injected hydrogel control groups. Furthermore, we assessed the *in vivo* growth plate cartilage repair effect among different groups to determine the ability of the 3D bioprinted scaffold to promote growth plate repair and inhibit deformities *in vivo*.

## 2 Materials and methods

### 2.1 Materials

PLGA (lactide/glycolide ratio 50:50, MW 65 kDa) and poly (vinyl alcohol) (PVA) (88%, MW 31–50 kDa) were purchased from Sigma-Aldrich (St. Louis, MO, United States). PCL (Polycaprolactone) was purchased from Macklin (Shanghai, China). Dulbecco’s Modified Eagle Medium (DMEM), and phosphate-buffered solution (PBS) were obtained from Gibco (Grand Island, United States). All reagents and chemicals were directly used without further treatment.

### 2.2 Preparation and characterization of PLGA microspheres

In this study, the w/o/w double emulsion technique was employed to create PLGA microspheres (Utzinger et al., 2017; Biswal et al., 2020). Specifically, 200 mg of PLGA was dissolved in 4 mL of dichloromethane, to which 500 μL of PTH(1–34) solution at a concentration of 100 μm and 150 μL of 6% gelatin were added. The mixture was stirred at 10,000 rpm for 1 min to generate an emulsion. The emulsion was introduced to 30 mL of 1% PVA solution and stirred at 8,000 rpm for 2 min. The emulsion was then gradually added dropwise to a three-necked flask containing 200 mL of 0.1% PVA solution and stirred at 1,000 rpm for 5 min. The resulting solution was transferred to a beaker and stirred slowly in a fume hood for 4 h to allow the dichloromethane to evaporate. Finally, the solution was centrifuged at 400 rpm to gather the microspheres, which were washed four times with deionized water and then lyophilized to acquire the PTH (1–34)-loaded microspheres.

The SEM technique was utilized to observe the morphology and particle size of the PLGA microspheres, and the particle size was assessed. For drug release studies, 100 mg of PTH(1–34) microspheres were dissolved in 20 mL of PBS solution and agitated at 37°C. At days 1, 7, 14, 21, 28, 42, and 56, 2 mL of the sample was taken and blended with 2 mL of fresh PBS, and the amount of PTH(1–34) released was quantified using the Enzyme-Linked Immunosorbent Assay (ELISA) to construct the release profile of PTH(1–34) *in vitro*.

### 2.3 Fabrication of composite hydrogels

Nucleated bone marrow mesenchymal stem cells (BMSCs) were isolated from femoral bone marrow of New Zealand white rabbits using density gradient centrifugation. Upon reaching 60% confluency, the cells were subcultured and maintained in a humidified incubator at 37°C with 5% CO_2_ until passage 3.

Gelatin methacryloyl (GelMA) was dissolved in PBS solution at 40°C to obtain a concentration of 100 mg/mL, with the addition of 0.25% (w/v) photoinitiator LAP. BMSCs and drug-loaded microspheres were encapsulated separately in the filter-sterilized hydrogel precursor solution at concentrations of 5 × 10^6^ cells/mL and 5 mg/mL, respectively. The precursor solution was UV-crosslinked to form the composite hydrogels for the purpose of fabricating the composite hydrogels.

### 2.4 Fabrication and characterization of 3D printed scaffolds

The 3D bioprinter (Bio-Architect, WS, China) was utilized to fabricate composite scaffolds by using the un-crosslinked hydrogel precursor solution as bioink. The biological printing process was conducted with the aid of a multi-nozzle and temperature control system. The high-temperature nozzle was used to deposit PCL at 80°C, which was then alternately printed on each layer. Meanwhile, the low-temperature nozzle was used to extrude bio-ink at 20°C, with a printing interval of 10 s between each layer, allowing for the photo-crosslinking of the bio-ink and cooling of the PCL. The printing parameters were set to produce semi-cylindrical scaffolds with a diameter of 6 mm, a height of 2 mm, and 20 layers in total, with a light intensity of 50 mW/cm^2^.

The scaffold groups were classified into three: (Yu et al., 2022): PG group: PCL + GelMA, (Tiffany and Harley, 2022), PGB group: PCL + GelMA + BMSCs, (Muruganandan et al., 2022), PGBP group: PCL + GelMA + BMSCs + PTH(1–34). The macroscopic morphology and microstructure of the scaffolds were characterized using optical microscopy and scanning electron microscopy, respectively. The compressive modulus of the scaffolds was assessed using a mechanical testing machine by conducting uniaxial compression tests at a stable strain rate of 1 mm/min, and the compressive modulus was calculated from the slope of the stress-strain curve at 0%–10% strain.

### 2.5 *In vitro* experiments of 3D printed scaffolds

Firstly, the viability and distribution of cells within the 3D printed composite scaffolds were evaluated. The scaffolds containing BMSCs were placed in a 24-well plate and cultured in DMEM medium at 37°C and 5% CO_2_ for 1 day. The scaffolds were then washed three times with PBS and stained with the live/dead staining solution, which was prepared by adding 4 nM calcein AM and 2 nM ethidium homodimer to PBS. After removing the staining solution, the scaffolds were washed twice with PBS and imaged using a confocal microscope. Secondly, BMSCs were co-cultured with the scaffold of PGB and PGBP group (5 × 10^3^/well) in a 96-well plate. The culture medium was removed on day 1, 3, and 7, and washed twice with PBS. After that, 90 μL of the medium and 10 μL of CCK-8 reagent were added to each well and incubated for 1 h in the dark. The absorbance values at 450 nm was measured with a microplate reader.

Finally, the chondrogenic differentiation of BMSCs in the 3D printed compoiste scaffolds was investigated. For the chondrogenic differentiation evaluation, the scaffolds were placed in a 24-well plate and cultured in DMEM medium at 37°C and 5% CO_2_ for 3 weeks. The medium was then removed, and the scaffolds were washed with PBS and fixed with paraformaldehyde. The scaffolds were stained with toluidine blue and Alcian blue, and the staining effect of the scaffolds was observed under a microscope.

### 2.6 *In vivo* experiment with growth plate defect animal model

All procedures complied with the Guide for the Care and Use of Laboratory Animals and were approved by the Ethics Committee. New Zealand White rabbits at 6 weeks of age, regardless of gender, were used for the experiment and randomly allocated into four groups: untreated group (blank group), GelMA group (GelMA hydrogel containing BMSCs without PLGA microspheres), PGB group (PCL + GelMA + BMSCs), and PGBP group (PCL + GelMA + BMSCs + PTH(1–34)).

Under anesthesia, the surgical site on the lower limb of the rabbit was disinfected with an iodine cotton ball after removing the hair. A longitudinal incision of approximately 2 cm was made from the medial gap of the knee joint to the inner side of the proximal tibia. The drilling position was selected as the exposed growth plate, and a horizontal defect with dimensions of 6 mm (length), 2 mm (width), and 3 mm (depth) was created by vertically drilling into the tibia at 10,000 rpm using an oral grinding drill bit. After the respective treatment based on the assigned groups, the rabbits were sutured and housed routinely post-surgery without immobilization. Daily injections of penicillin were administered for 3 days following the surgery. The rabbits were euthanized at 4 and 12 weeks postoperatively, and the proximal tibia specimens were macroscopically photographed, X-ray imaged, and micro-CT scanned. The specimens were sectioned for histological analysis and stained with HE to evaluate the regeneration and repair of the growth plate cartilage in each group.

### 2.7 Statistical analysis

Quantitative data are presented as the mean ± standard deviation (Mean ± SD). Multi-group comparisons were analyzed by oneway analysis of variance (ANOVA) with the Tukey test. Statistical analysis was performed using GraphPad Prism 8.0. *p* < 0.05 is considered to be statistically significant.

## 3 Results

### 3.1 Preparation and characterization of PLGA microspheres

**FIGURE 2:**
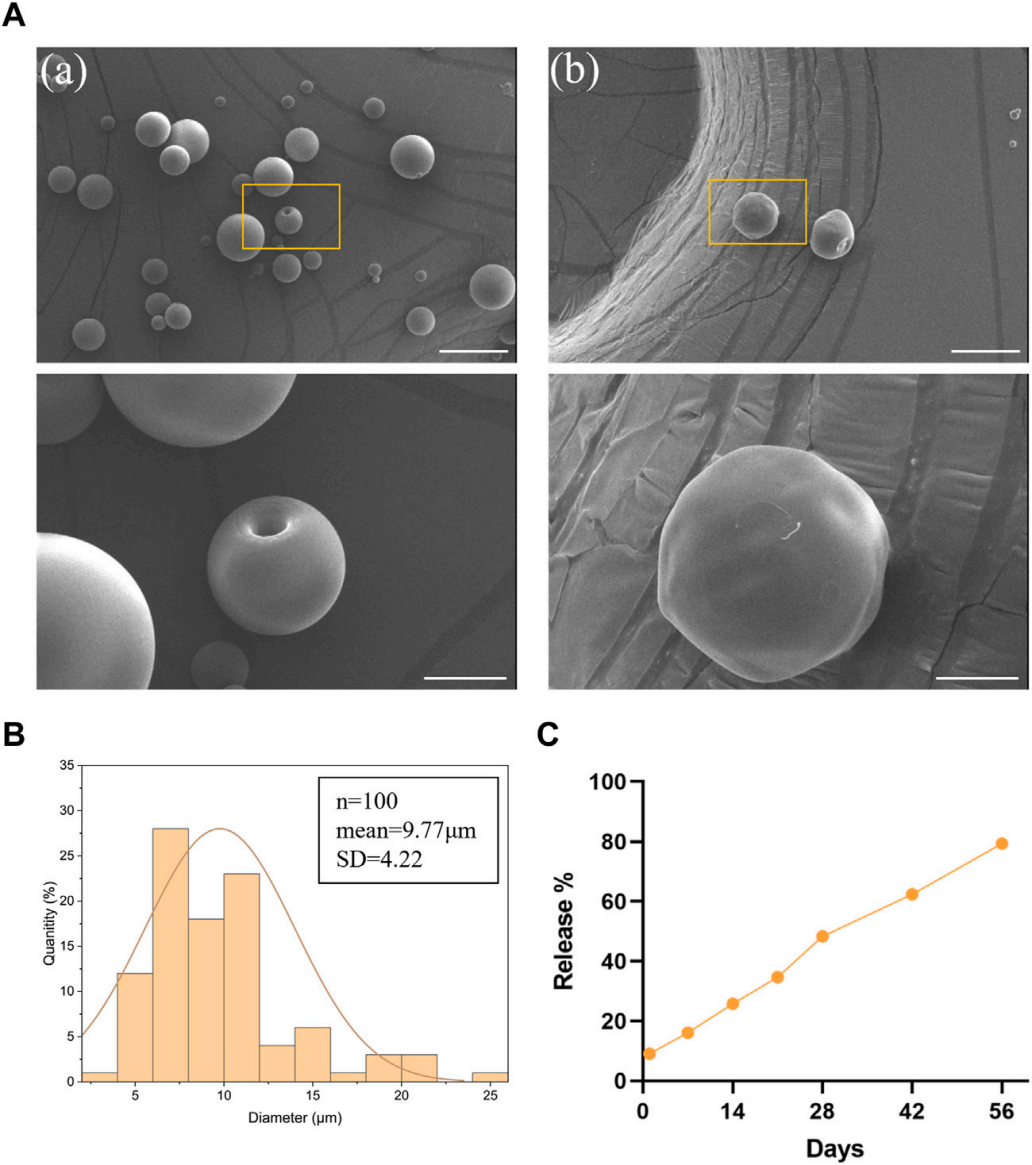
Preparation and characterization of PLGA microspheres. **(A)** SEM images of blank (a) and PTH(1–34)-loaded (b) PLGA microspheres. Scale bar on top row of images is 20 μm and higher-resolution images (bottom row) scale bar represents 5 μm. **(B)** particle size distribution of PLGA microspheres. **(C)** The *in vitro* release profile of the loaded PTH(1–34) in the microspheres.

In this study, the blank and PTH(1–34)-loaded PLGA microspheres were prepared and characterized. The morphology of the microspheres was evaluated using scanning electron microscopy, and both types of microspheres were found to have intact shapes with smooth surfaces, without any adhesion (Figure 2A). The average diameter of the microspheres was determined to be 9.77 ± 4.22 μm (Figure 2B), indicating minimal size variation within the population. The drug release profile of the PTH(1–34)-loaded PLGA microspheres showed a slow and sustained release pattern, with no burst release observed. By day 56, the cumulative release of PTH(1–34) from the microspheres reached approximately 80%, indicating a favorable sustained release behavior (Figure 2C).

### 3.2 Fabrication and characterization of 3D printed scaffolds

**FIGURE 3:**
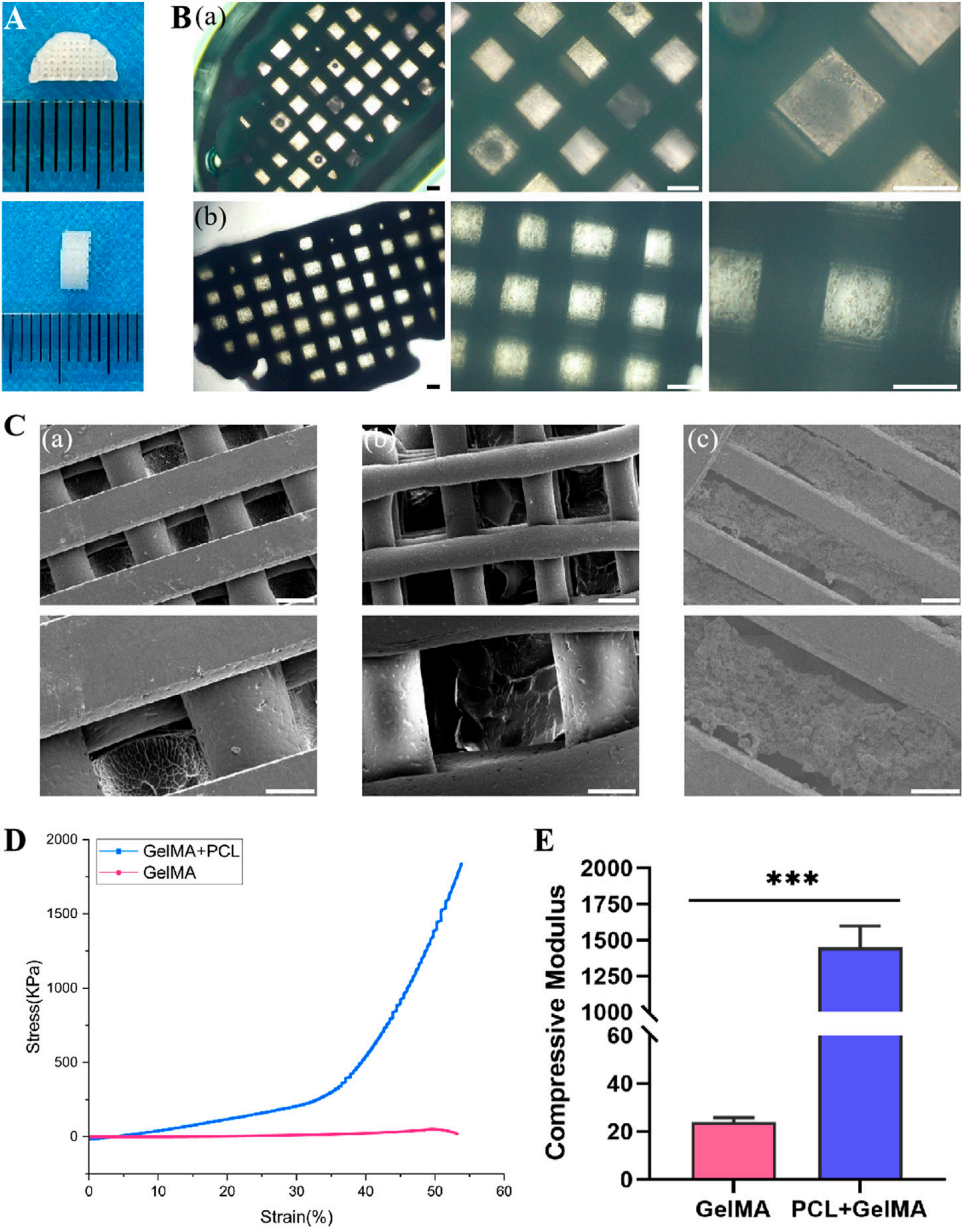
Fabrication and characterization of 3D printed scaffolds. **(A)** Gross appearance of 3D printed scaffolds. **(B)** Light microscopic images of scaffolds in PGB group (a) and PGBP group (b). Scale bar represents 200 μm. **(C)** SEM images of scaffolds in PG group (a), PGB group (b) and PGBP group (c). Scale bar on the first column of images is 200 μm and higher-resolution images (the second column) scale bar represents 100 μm. **(D)** Representative stress-strain curves of the GelMA hydrogel and 3D printed scaffold. **(E)** Quantitative analysis of Young’s modulus of compression.

A precursor solution for the composite hydrogel was created by encapsulating PLGA microspheres and BMSCs within GelMA hydrogel, which was then combined with PCL via 3D bioprinting technology to fabricate the scaffold. The overall architecture of the 3D printed composite scaffold was semi-cylindrical, with a height of 2 mm and a diameter of 6 mm, as depicted in Figure 3A. Upon microscopic examination, the scaffold structure displayed excellent connectivity and precise, ordered arrangement, as demonstrated in Figure 3B. The scanning electron microscopy data revealed that the scaffold surface contained regular pores and well-defined PCL structures. The hydrogel was distributed evenly in the gaps between the PCL, encapsulating the PLGA microspheres and BMSCs, as depicted in Figure 3C.

Given that GelMA hydrogel’s mechanical strength is typically low, the addition of PCL can augment its mechanical properties. As demonstrated in Figures 3D,E, the stress-strain curves of each group’s scaffolds were analyzed, and the compressive modulus was calculated from the slope of the stress-strain curve between 0% and 10% strain. The compressive modulus of the composite hydrogel was found to be relatively low, measuring 23.85 kPa. However, the addition of PCL resulted in a substantial increase in compressive modulus, reaching approximately 1452 kPa, respectively (Figure 3E).

### 3.3 The 3D-printed scaffold has good biocompatibility and promotes chondrogenic differentiation

**FIGURE 4:**
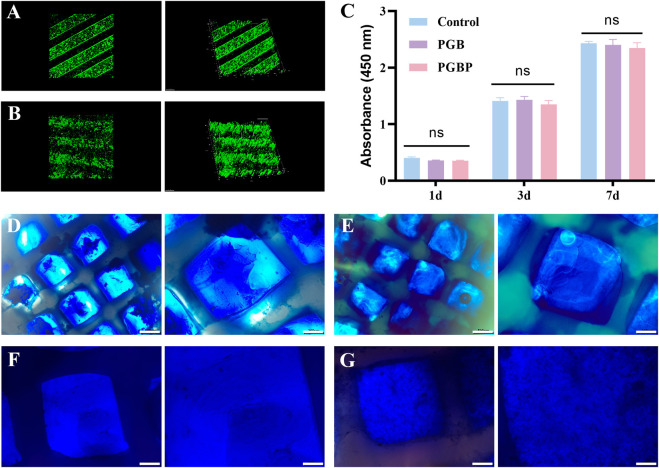
3D printed scaffolds supported the viability and chondrogenic differentiation of BMSCs. **(A–B)** Representative live/dead staining of BMSCs was performed for the scaffolds in PGB group **(A)** and PGBP group **(B)** after 1 day of culture. **(C)** Cell proliferation of BMSCs cultured on the scaffold of each group after culturing for 1, 3, and 7 days. **(D–E)** Representative images from Alcian blue staining for the scaffolds in PGB group **(C)** and PGBP group **(D)** after 3 weeks of culture. Scale bar represents 500 μm (left) and 200 μm (right). **(F–G)** Representative images from Toluidine blue for the scaffolds in PGB group **(E)** and PGBP group **(F)** after 3 weeks of culture. Scale bar represents 200 μm (left) and 100 μm (right).

Following 24 h of culture in DMEM medium, the scaffolds comprising BMSCs were subjected to live/dead staining to assess the cell viability within the scaffold. The results demonstrated that the BMSCs within the scaffold of each group displayed good cell viability and were uniformly distributed, indicating excellent biocompatibility of the scaffold (Figures 4A,B). In addition, the biocompatibility of 3D printed scaffolds was evaluated by co-culturing them with BMSCs, and their effects on cell proliferation were determined using the CCK-8 assay. The results showed that there were no evident differences regarding the proliferative rate of the BMSCs among the control, PGB and PGBP groups (Figure 4C), indicating that the scaffolds exerted good biocompatibility. To further evaluate the chondrogenic differentiation of BMSCs in the scaffold, alcian blue and toluidine blue staining were carried out after 3 weeks of culture. The cartilaginous matrix produced by BMSCs co-cultured with the scaffold was evaluated through staining with alcian blue and toluidine blue. The results demonstrated that the PGBP group exhibited better chondrogenic effects compared to the PGB group (Figures 4D–G). The BMSCs in the PGBP group yielded a cartilaginous matrix that stained positively for toluidine blue and alcian blue, indicating a proteoglycan-rich, cartilage-like extracellular matrix. These findings suggest that the addition of PTH(1–34) to the PGB scaffold promoted chondrogenic differentiation of BMSCs. This demonstrates the potential of the PTH(1–34)@PLGA/BMSCs/GelMA-PCL scaffold as a promising approach for the repair of injured growth plate.

### 3.4 *In vivo* animal experiments of scaffolds and injectable hydrogels

**FIGURE 5:**
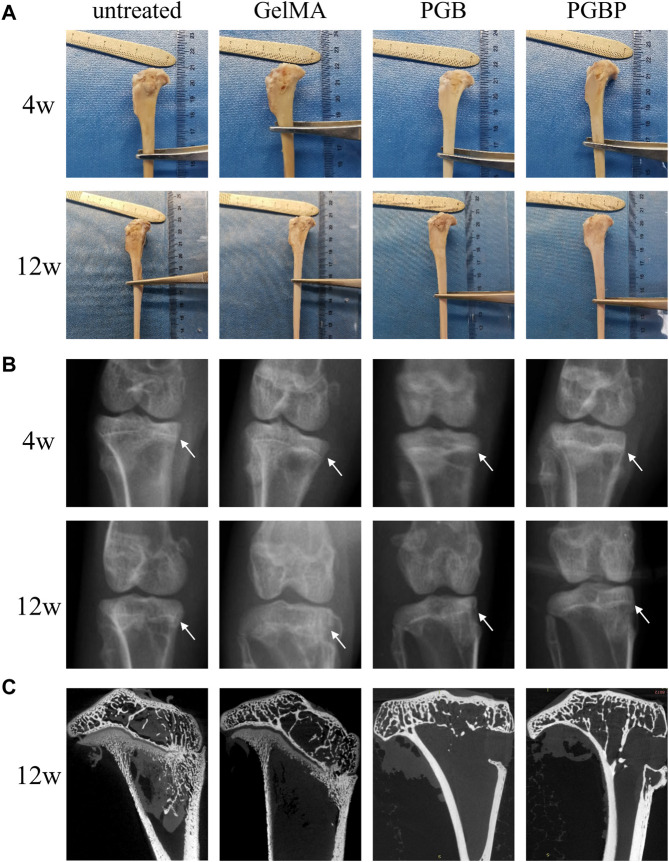
3D printed scaffolds promoted the regeneration of growth plate cartilage in the rabbit model. **(A)** Gross appearance of the injured growth plate of tibia in each group at 4 weeks and 12 weeks postoperatively. **(B)** X-ray images of the injured growth plate of tibia in each group at 4 weeks and 12 weeks postoperatively. The site of growth plate injury is indicated by white arrows. **(C)** Micro-CT images of the injured growth plate of tibia in each group at 12 weeks postoperatively. And the defect site is displayed on the coronal-section. Scale bar represents 5 mm.

New Zealand white rabbits were employed as experimental models to assess the regenerative capacity of each group on injured growth plates. The macroscopic images showed no significant improvement in the gross appearance of the tibial growth plate injury site in the GelMA and PGB groups compared to the untreated group, and in some cases, obvious bone tissue formation was observed (Figure 5A). Conversely, the PGBP group demonstrated improved gross appearances, and new cartilage tissue was evident at the defect site. Moreover, the X-ray exhibited no significant variation in calcified tissue between the hydrogel and PGB groups relative to the untreated group, whereas there was a reduction in calcified tissue in the PGBP group (Figure 5B). The micro-CT images revealed that at the defect site of tibia growth plate, obvious overall collapsed and angular deformities were present in the untreated group and GelMA group, while the PGB and PGBP group had a lesser degree or even no deformities (Figure 5C). This finding emphasized the crucial role of the mechanical support provided by scaffold in promoting the repair of growth plate injuries.

**FIGURE 6:**
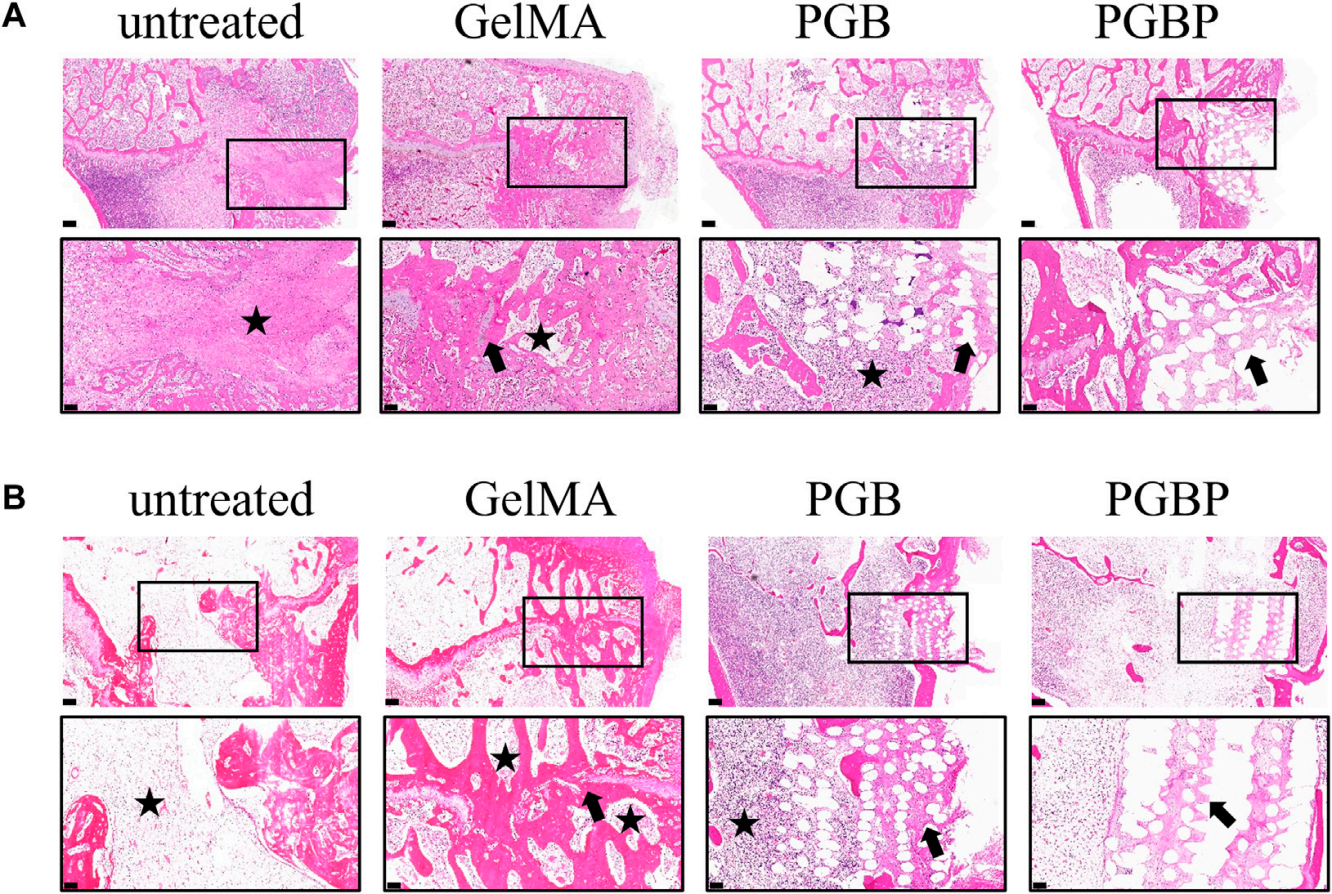
3D printed scaffolds promoted the repair of growth plate cartilage *in vivo*. **(A–B)** HE staining of histological sections of the injured growth plate of tibia in each group at 4 weeks **(A)** and 12 weeks **(B)** postoperatively. The scale bar of 500 μm is used to represent the original magnification, while the boxed area, which represents the defect site, is further enlarged and shown below with a scale bar of 200 μm. The black star symbol marks the bone tissue, and the black arrow marks the regenerated cartilage tissue.

Histological sections indicated that the untreated group (control group) had minimal new cartilage tissue, with a significant amount of bone tissue at the site of growth plate injury (Figure 6). In the GelMA group, a small amount of regenerated cartilage tissue was observed, but it was still predominantly bone tissue with slight collapsed deformity. The PGB group did not display significant tissue collapse or deformity, which could be attributed to the implantation of scaffold, although there was little new cartilage tissue formation within the scaffold. Finally, the PGBP group exhibited more new cartilage tissue than the other groups, and no obvious signs of deformity were observed. The results suggest that the 3D-printed PGBP scaffold provides an ideal mechanical support for the growth plate and enhances chondrogenic differentiation of BMSCs, leading to the efficient regeneration of growth plate cartilage.

## 4 Discussion

The growth plate is prone to injury, which can result in bone bridges, causing limb length discrepancy, angular deformity, and other symptoms such as gait disturbance, back pain, and early-onset osteoarthritis (Agirdil, 2020). Current tissue engineering strategies for treating growth plate injuries mainly focus on seed cells or cytokines (Chung and Xian, 2014), while the role of tissue engineering scaffolds in providing mechanical support is often overlooked. This study aims to use 3D bioprinting technology to create a tissue engineering scaffold that can promote the repair of growth plate injuries by considering both cytokines and scaffold structure.

The growth plate is divided into four distinct zones from the distal (epiphysis) to the proximal (metaphysis) end, including the resting, proliferation, hypertrophic, and ossification zones (Agirdil, 2020). Chondrocytes in the proliferation zone rapidly divide and align into columns parallel to the growth direction. Then, these chondrocytes exit the cell cycle and further differentiate into hypertrophic chondrocytes, forming the hypertrophic zone. Hypertrophic chondrocytes in the hypertrophic zone undergo apoptosis or transdifferentiate into osteoblasts, gradually forming new bone tissue, thereby playing a biological role in limb lengthening. Thus, cellular activity in the proliferation zone plays a critical role in growth plate cartilage regeneration (Villemure and Stokes, 2009; Myllyharju, 2014). The PTHrP-Ihh signaling axis is a vital negative feedback loop that regulates chondrocyte proliferation and hypertrophy in the growth plate. PTHrP is secreted by resting zone chondrocytes and diffuses down the growth plate to promote chondrocyte proliferation and delay hypertrophy through binding to PTH/PTHrP receptors on proliferating chondrocytes, which is crucial for growth plate cartilage regeneration PTH(1–34), as an exogenous PTHrP activator, can promote chondrogenesis and chondrocyte proliferation by regulating PTHrP-Ihh signaling axis signal transduction (Erickson et al., 2018). Therefore in this study, PTH(1–34) was selected as the cytokine to be incorporated into the tissue engineering scaffolds.

Considering the prolonged and continuous process required for the repair of growth plate, it is essential for drug factors to maintain their effective concentration at the site of injury to exert their intended effects. To achieve this objective, this study employed microspheres to achieve sustained release of drug factors. PLGA microspheres are a commonly used drug release system that enables the slow and sustained release of loaded drugs. Therefore, PTH(1–34) was loaded into PLGA microspheres to achieve sustained drug release. *In vitro* drug release curve results indicated that PTH(1–34) loaded PLGA microspheres exhibited a sustained drug release pattern without any significant burst release phenomenon, with a release duration of more than 3 weeks. Furthermore, *in vitro* cell experiments revealed that PTH(1–34) induced BMSCs to differentiate into chondrocytes. The HE stained images of histological slices also demonstrated that the PGBP group containing PTH(1–34) exhibited more visible regeneration of cartilage-like tissue compared to the untreated and PGB group. The PTH(1–34)@PLGA/BMSCs/GelMA-PCL scaffold demonstrated a positive impact on the repair of growth plate injuries, as indicated by the improved chondrogenic differentiation of BMSCs and regeneration of growth plate cartilage in the PGBP group compared to the untreated and PGB group. It is a fact that the repair of injured growth plate could be attributed to the *in vivo* action of various cytokines and the complex regulatory signaling pathways involved. Therefore, further investigations into the signaling regulation mechanisms in the growth plate are necessary to determine the role of the hydrogel scaffold in promoting *in vivo* cartilage regeneration. Additionally, growth plate chondrocytes play a crucial role in the biological process of cartilage endochondral ossification, which is a crucial mechanism for growth plate to promote bone growth. However, it remains unclear whether regenerated chondrocytes have the same biological function as uninjured chondrocytes. Therefore, future studies need to focus on functional regeneration in addition to the histological regeneration of growth plate.

Following growth plate injury, the formation of bone bridge results in the loss of growth plate function, leading to limb length discrepancy and deformities (Sanghani et al., 2013). Implant materials aim to prevent bone bridge formation, but studies on tissue engineering composite materials and gels have revealed some degree of bone filling and growth plate closure in these treatments (Chen et al., 2003; Jin et al., 2006; Sundararaj et al., 2015), making it difficult to determine whether the mineralization of the defect area was normal bone closure or bone bridge remodeling. Injectable hydrogels such as agarose, collagen, and hyaluronic acid have been used to prevent the formation of bone bridge in previous studies, but their elastic modulus ranged from 0.001–0.150 Mpa (Tobita et al., 2002; Chen et al., 2003; Coleman et al., 2010), which is far lower than that of natural tissues that reported. To promote the regeneration of growth plate cartilage and provide good mechanical support, a scaffold with mechanical properties similar to those of natural tissues is required 0.48 Mpa (Eckstein et al., 2022; Yu et al., 2022). Yu et al. demonstrated that the treatment of bone bridge excision combined with a 3D printed growth plate simulation composite scaffold led to significantly more limb growth within 8 weeks compared to other treatments (Yu et al., 2022). This may be due to the 3D printed scaffold acting as a gap material that limits bone bridge formation and provides better mechanical support. Therefore, it is crucial to develop scaffolds with appropriate mechanical properties similar to those of natural growth plate to promote growth plate cartilage regeneration and prevent bone bridge formation.

Although its promising potential, GelMA still falls short of the mechanical strength required for mimicking natural growth plates. Hence, PCL was incorporated into the 3D printing process to bolster the overall mechanical properties of the scaffold. Although PCL has modest biological activity, it has excellent biocompatibility, biodegradability, and mechanical strength, which qualifies it as a clinically feasible biomaterial authorized by the FDA (Siddiqui et al., 2018; Smaida et al., 2020). Mechanical testing demonstrated that the inclusion of PCL significantly enhanced the scaffold’s compressive modulus, aligning it more closely with that of the natural growth plate. Likewise, *in vivo* results revealed that the effect of cartilage regeneration in the scaffold was superior to that of the injectable hydrogel, with no apparent cartilage tissue collapse. This suggests that the scaffold’s mechanical support can not only prevent bone invasion caused by cartilage tissue collapse and minimize bone bridge formation but also provide the necessary space for essential regenerative activities, such as cell adhesion, proliferation, differentiation, and collagen deposition. However, this study failed to observe any significant limb elongation, and the outcome was consistent with previous finding of a lack of significant endochondral ossification. It remains unclear whether the scaffold’s additional mechanical support or the synergistic effect of the scaffold and cartilage simulated hydrogel filling contributed to tibial elongation (Yu et al., 2022). Hence, the microstructure of scaffolds may also play a crucial role in this regard, such as gradient pore size scaffold or multilayered scaffold, which simulate the microstructure and layering of natural growth plate. Nonetheless, the study did manage to improve the angle deformity after growth plate injury to a certain degree, hinting at the potential of scaffold to restore the growth plate’s growth function and avoid limb deformity. Further investigations into the microstructure of growth plate cartilage tissue and determine the scaffold microstructure’s role in promoting growth plate cartilage regeneration.

To sum up, our study utilized 3D printing technology to fabricate a PCL-GelMA scaffold incorporating PTH(1–34) microspheres and BMSCs. The scaffold exhibited an interconnected porous network structure, favorable biocompatibility, and mechanical properties that supported cell differentiation. We demonstrated that the scaffold was more effective in promoting cartilage tissue regeneration and inhibiting bone bridge formation compared to the direct injection of hydrogel *in vivo*. These findings are valuable in advancing growth plate cartilage tissue engineering approaches and offer potential applications in biological implantation for treating growth plate injuries.

## Data Availability

The original contributions presented in the study are included in the article/supplementary material, further inquiries can be directed to the corresponding author.
